# Supplementary material for: Dietary ω−3 polyunsaturated fatty acids (PUFAs) reduce cholesterol-driven non-small cell lung cancer (NSCLC) progression in mouse models of disease
Source: Commun Med (Lond). 2025 Oct 23;5:432. doi: 10.1038/s43856-025-01193-y (PMC12549838; doi:10.1038/s43856-025-01193-y)
Supplement: Supplementary file 2 — Supplementary Information [file 43856_2025_1193_MOESM2_ESM.pdf]

Supplementary Information

Dietary ω-3 polyunsaturated fatty acids (PUFAs) reduce cholesterol-driven non-small cell lung cancer (NSCLC) progression in mouse models

Philipp Harre[1, *], Katja Hohenberger[1], Susanne Krammer[1], Zuqin Yang[1], Patrick Tausche[1], Jonas Willar[1], Denis I. Trufa [2,3,4], Mircea T. Chiriac [5], Carol I. Geppert [3,4,6.], Manfred Rauh[7], Kai Hildner [3.4,5], Arndt Hartmann [3,4,5], Horia Sirbu[2,3,4] and Susetta Finotto [1,3,4,8**]

Philipp Harre[1,*], Katja Hohenberger[1,§], Susanne Krammer[1,§], Zuqin Yang[1,§], Patrick Tausche[1,§], Denis I. Trufa [2,3,4], Kai Hildner [3.4,5], Mircea T.Chiriac [5], Carol I. Geppert [3,4,6.], Manfred Rauh[7], Arndt Hartmann [3,4,5], Horia Sirbu[2,3,4] and Susetta Finotto [1,3,4,8**]

[1] Department of Molecular Pneumology, Friedrich-Alexander-Universität (FAU) Erlangen-Nürnberg, Universitätsklinikum Erlangen, 91052 Erlangen, Germany
[2] Department of Thoracic Surgery, Friedrich-Alexander-Universität (FAU) Erlangen-Nürnberg, Universitätsklinikum Erlangen, 91052 Erlangen, Germany
[3] Bavarian Cancer Research Center (BZKF), Erlangen, Germany,
[4] Comprehensive Cancer Center Erlangen-EMN (CCC ER-EMN), Erlangen, Germany
[5] Department of Medicine 1 - Gastroenterology, Pneumology and Endocrinology, Friedrich-Alexander-Universität (FAU) Erlangen-Nürnberg, Universitätsklinikum Erlangen, 91052 Erlangen, Germany
[6] Institute of Pathology, Friedrich-Alexander-Universität (FAU) Erlangen-Nürnberg, Universitätsklinikum Erlangen, 91052 Erlangen, Germany
[7]Department of Paediatrics and Adolescent Medicine, Friedrich-Alexander-Universität (FAU) Erlangen-Nürnberg, Universitätsklinikum Erlangen, 91052 Erlangen, Germany
[8] Deutsches Zentrum für Immuntherapie (DZI), Erlangen, Germany,
*The present work was performed in fulfilment of the requirements for obtaining the degree "Dr. med." for Philipp Harre

§Contributed equally to this work

**Corresponding author

Prof. Susetta Finotto, PhD
Laboratories of Cellular and Molecular Lung Immunology
Department of Molecular Pneumology
Friedrich-Alexander-Universität Erlangen-Nürnberg
Hartmannstraße 14
91052 Erlangen, Germany
Phone: 0049-9131-8535883
Email: Susetta.Finotto@uk-erlangen.de

Supplementary Table 1: Individual clinical data of our cohort.

Patient Code	Age	Gender	Body weight [kg]	Body height [m]	BMI [Weight/Height^2]	Waist circumference [cm]
NSCLC001	80-84	Female	75	1.58	30.04	116
NSCLC002	60-64	Male	95	1.74	31.38	105
NSCLC003	70-74	Male	78	1.82	23.55	98
NSCLC004	50-54	Female	73	1.6	28.52	-
NSCLC005	50-54	Female	57	1.68	20.20	-
NSCLC006	70-74	Female	85	1.66	30.85	102
NSCLC007	50-54	Male	120	1.87	34.32	112
NSCLC008	60-64	Male	80	1.8	24.69	-
NSCLC009	60-64	Male	113	1.91	30.98	-
NSCLC010	60-64	Female	75	1.6	29.30	-
NSCLC011	80-84	Female	125	1.65	45.91	148
NSCLC012	40-44	Male	82	1.76	26.47	-
NSCLC013	75-79	Female	63	1.58	25.24	-
NSCLC014	60-64	Female	68	1.68	24.09	-
NSCLC015	60-64	Male	108	1.76	34.87	112
NSCLC016	60-64	Male	79	1.74	26.09	-
NSCLC017	60-64	Female	57	1.56	23.42	91
NSCLC018	65-69	Male	72	1.73	24.06	90
NSCLC019	70-74	Male	103	1.88	29.14	109
NSCLC020	55-59	Female	43	1.58	17.22	76
NSCLC021	60-64	Male	56	1.72	18.93	-
NSCLC022	65-69	Male	70	1.83	20.90	93
NSCLC023	70-74	Female	62	1.59	24.52	-
NSCLC024	75-79	Male	73	1.68	25.86	-
NSCLC025	75-79	Male	69	1.68	24.45	89
NSCLC026	50-54	Female	55	1.6	21.48	-
NSCLC027	60-64	Female	50	1.58	20.03	-
NSCLC028	75-79	Female	64	1.58	25.64	100
NSCLC029	70-74	Female	78	1.63	29.36	110
NSCLC030	65-69	Female	83	1.58	33.25	-
NSCLC031	65-69	Female	68	1.57	27.59	-
NSCLC032	65-69	Male	75	1.82	22.64	-
NSCLC033	60-64	Male	85	1.79	26.53	-

Supplementary Table 2: Individual clinical data of our cohort.

(LUAD = lung adenocarcinoma; y = years)

Patient Code	Histological Classification	Grading	TNM-Stadium	Tumor Diameter [cm]	Smoking (pack years)
NSCLC001	LUAD	G2	IIIA	2.7	0
NSCLC002	LUAD	G3	IA3	2.5	100
NSCLC003	LUAD	G2	IIA	4.5	75
NSCLC004	LUAD	G3	IVA	1.3	50
NSCLC005	LUAD	G2	IA2	1.8	45
NSCLC006	LUAD	G3	IIIA	4	second hand smoker (25y)
NSCLC007	LUAD	G1	IA2	1.5	70
NSCLC008	LUAD	G3	IVA	2.3<br>2.5	0
NSCLC009	LUAD	G2	IA2	1.4	30
NSCLC010	LUAD	G3	IIIA	1.8	40
NSCLC011	LUAD	G2	IB	3.5	0
NSCLC012	LUAD	G3	IIIA	8.5	22
NSCLC013	LUAD	G2	IB	3.1	0
NSCLC014	LUAD	G2	IA1	0.9	45
NSCLC015	LUAD	G3	IIB	5.4	28
NSCLC016	LUAD	G3	IA2	1.6	46
NSCLC017	LUAD	G3	IVA	5.5	0
NSCLC018	LUAD	G2	IIIA	3.6	0
NSCLC019	LUAD	G2	IIB	5	0
NSCLC020	LUAD	G3	IIIA	2.9	30
NSCLC021	LUAD	G3	IIIA	3.5<br>1.5	0
NSCLC022	LUAD	G2	IIB	2.1	18
NSCLC023	LUAD	G3	IB	3.9	100
NSCLC024	LUAD	G3	IA2	1.8	35
NSCLC025	LUAD	G2	IA2	1.2	60
NSCLC026	LUAD	G1	IA2	1.7	20
NSCLC027	LUAD	G2	IB	3.6	40
NSCLC028	LUAD	G3	IIIB	7.2	45
NSCLC029	LUAD	G2	IIIA	1.5<br>1.6	0
NSCLC030	LUAD	G1	IA2	1.5	50
NSCLC031	LUAD	G1	IA	1.8	40
NSCLC032	LUAD	G2	IA2	1.2	84
NSCLC033	LUAD	G1	IIA	4.2	15

Supplementary Table 3: Individual clinical data of our cohort.

Patient Code	Glucose [mg/dl]	Total cholesterol presurgical [mg/dl]	HDL cholesterol presurgical [mg/dl]	LDL cholesterol presurgical [mg/dl]	Triglycerides presurgical [mg/dl]	Lipoprotein A presurgical [mg/dl]
NSCLC001	83.00	-	-	-	-	-
NSCLC002	125.00	-	-	-	-	-
NSCLC003	101.00	-	-	-	-	-
NSCLC004	104.00	-	-	-	-	-
NSCLC005	84.00	-	-	-	-	-
NSCLC006	96.00	202.00	72.00	113.00	83.00	38.70
NSCLC007	108.00	107.00	32.00	58.00	115.00	7.90
NSCLC008	96.00	151.00	23.00	105.00	161.00	7.90
NSCLC009	101.00	156.00	32.00	86.00	139.00	7.90
NSCLC010	92.00	222.00	64.00	135.00	105.00	14.20
NSCLC011	89.00	138.00	37.00	81.00	147.00	7.90
NSCLC012	91.00	115.00	27.00	72.00	87.00	7.90
NSCLC013	81.00	-	-	-	-	-
NSCLC014	259.00	208.00	61.00	127.00	57.00	7.90
NSCLC015	106.00	206.00	27.00	141.00	204.00	7.90
NSCLC016	98.00	151.00	34.00	97.00	100.00	7.90
NSCLC017	98.00	152.00	38.00	92.00	115.00	7.90
NSCLC018	101.00	180.00	83.00	80.00	72.00	30.20
NSCLC019	127.00	150.00	29.00	98.00	188.00	7.90
NSCLC020	102.00	206.00	99.00	86.00	87.00	11.70
NSCLC021	-	174.00	34.00	117.00	115.00	19.30
NSCLC022	101.00	152.00	38.00	96.00	84.00	13.90
NSCLC023	108.00	208.00	61.00	118.00	139.00	66.00
NSCLC024	-	180.00	61.00	90.00	180.00	7.90
NSCLC025	-	151.00	41.00	90.00	112.00	7.90
NSCLC026	97.00	296.00	66.00	186.00	173.00	7.90
NSCLC027	116.00	217.00	48.00	141.00	98.00	9.70
NSCLC028	-	170.00	57.00	94.00	106.00	13.10
NSCLC029	101.00	178.00	41.00	108.00	77.00	7.90
NSCLC030	-	253.00	55.00	157.00	182.00	15.70
NSCLC031	101.00	183.00	63.00	108.00	77.00	7.90
NSCLC032	109.00	165.00	80.00	72.00	94.00	18.70
NSCLC033	98.00	167.00	47.00	103.00	169.00	94.70

Supplementary Table 4: Individual clinical data of our cohort.

Patient Code	Albumin presurgical[g/l]	CRP presurgical [mg/l]	CRP postsurgical [mg/dl]	Lymphocytes total presurgical [x10^3/µl]	Lymphocytes presurgical [%]	FEV1 [%]	FVC [%]
NSCLC001	38.80	2.00	62.40	2.14	29.90	85.50	98.20
NSCLC002	-	24.40	183.80	1.93	15.10	96.50	76.80
NSCLC003	41.90	2.60	159.20	1.49	21.30	116.80	118.20
NSCLC004	39.80	1.50	73.00	1.08	13.00	105.80	114.10
NSCLC005	45.20	-	58.20	1.59	30.20	40.10	90.00
NSCLC006	41.50	1.50	25.20	1.21	20.80	94.40	97.30
NSCLC007	45.50	0.80	66.90	2.13	30.60	87.80	85.90
NSCLC008	38.00	-	3.40	2.32	26.20	76.30	87.20
NSCLC009	43.80	5.10	93.90	2.32	29.40	52.80	65.70
NSCLC010	42.80	3.00	78.60	3.51	39.80	82.00	98.60
NSCLC011	28.50	8.80	142.30	1.78	31.00	100.20	-
NSCLC012	37.00	130.00	43.10	1.77	24.70	103.70	96.30
NSCLC013	44.60	2.30	114.70	1.36	23.10	97.40	101.00
NSCLC014	45.80	2.40	47.30	2.00	31.20	96.60	102.00
NSCLC015	41.30	9.00	239.20	1.63	18.50	62.80	63.80
NSCLC016	41.20	1.10	73.20	3.27	48.70	102.90	115.80
NSCLC017	45.30	9.70	201.60	1.14	14.70	113.60	105.50
NSCLC018	46.30	0.80	180.80	1.15	17.20	-	-
NSCLC019	43.50	<0.2	75.20	1.91	32.90	94.10	103.20
NSCLC020	42.90	0.60	44.20	2.10	27.00	74.80	97.10
NSCLC021	39.30	38.80	394.80	1.07	6.00	68.90	103.70
NSCLC022	38.20	6.20	190.70	2.70	36.70	-	-
NSCLC023	41.50	1.10	105.30	2.01	28.20	81.20	110.60
NSCLC024	-	-	74.50	0.49	9.40	64.60	-
NSCLC025	-	-	111.10	-	-	65.50	86.80
NSCLC026	45.80	2.60	229.70	2.91	25.50	-	-
NSCLC027	46.90	9.00	44.30	2.62	21.30	74.00	-
NSCLC028	-	56.10	125.40	1.26	9.10	95.70	86.40
NSCLC029	42.30	2.80	113.10	2.52	32.30	88.90	87.10
NSCLC030	44.30	5.4	31.50	2.07	22.50	87.10	100.40
NSCLC031	39.90	1.6	28.80	1.43	25.40	80.00	94.00
NSCLC032	38.10	0.9	30.90	1.24	15.10	40.00	57.00
NSCLC033	-	0.3	58.4	1.46	30.60	99.00	105.00

Supplementary Table 5: Micro nutrition diet of the mice

Diet	standard (breeding) diet [SD]	arteriosclerosis diet [AD]	omega-3 rich diet [OD]
**Trace elements [mg/kg]**			
Aluminium	79.37	2.696	12.001
Chlorine	3484.07	1602.307	3630
Iron	192.51	47.341	204.626
Flourine	2.8	6.271	4.17
Iodine	1.66	0.241	0.45
Cobalt	0.34	0.019	0.133
Copper	12.81	6.363	8.256
Manganese	95.06	53.086	102.701
Molybdenum	1.140	0.025	0.198
Sulfur	1141.22	2089.695	2486.775
Selenium	0.25	0.212	0.235
Zinc	95.18	36.953	31.436
**Added vitamins**			
Biotin [µg/kg]	167	472	200
Choline chloride [mg/kg]	600	7.188	1000
Folic acid [mg/kg]	2	1.9993	10
Nicotinic acid [mg/kg]	36	99.32	50
Pantothenic acid [mg/kg]	21	66.194	50
Vitamine A [IU/kg]	15000	20044	15000
Vitamine B1 [mg/kg]	18	22	20
Vitamine B2 [mg/kg]	12	22	20
Vitamine B6 [mg/kg]	9	22	15
Vitamine B12 [µg/kg]	24	37	30
Vitamine C [mg/kg]	36	1000	20
Vitamine D3 [IU/kg]	600	2204	500
Vitamine E [mg/kg]	75	120	150
Vitamine K3 [mg/kg]	3	25	10

Supplementary Table 6: Micro nutrition mice diet

Diet	standard (breeding) diet [SD]	arteriosclerosis diet [AD]	omega-3 rich diet [OD]
**Fatty acids [mg/kg]**			
Arachidic acid C-20:0	35	1444	1375
Eicosanoic acid C-20:1	44	614	2525
α-Linolenic acid C-18:3	2210	1846	14655
Linolenic acid C-18:2	16152	11266	32507
Palmitic acid C-16:0	3878	34635	12347
Stearic acid C-18:0	1187	29196	4590
Oleic acid C-18:1	6823	54988	129568
**Amino acids [g/kg]**			
Alanine	8.2	5.7	8.3
Arginine	11.1	10.3	12
Aspartic acid	15.3	5.8	21.1
Cystine	3.2	2.1	2.9
Glutamic acid	38.7	22.3	43.8
Glycine	8	10.4	7
Histidine	4.3	4	4.8
Isoleucine	7.4	5.8	9
Leucine	13.2	11.7	10
Lysine	8.1	13.5	5.6
Methionine	2.7	8.1	2.9
Phenylalanine	8.2	6.1	6.6
Proline	12.5	14.2	8.1
Serine	8.9	5.4	8.9
Threonine	6.4	5.8	7
Tryptophan	2.3	1.3	2.9
Tyrosine	5.8	6.4	6.4
Valine	8.6	5.8	4.68

Supplementary Table S7: Cell culture media used for human and murine experiments

Medium	Ingredients
RPMI medium without supplements	Gibco™ RPMI 1640 Medium, Thermo Fisher Scientific (cat# 21875091)
RPMI medium	Gibco™ RPMI 1640 Medium, Thermo Fisher Scientific (cat# 21875091); 50 ml heat-inactivated fetal bovine serum (FCS), Sigma-Aldrich (cat# S0615); 2mM L-Glutamine, anprotec (cat# AC-AS-0001); 5 ml Penicillin-Streptomycin (Pen/Strep), anprotec (cat# AC-AB-0024)
DMEM medium without supplements	500 ml GibcoTM DMEM, high glucose, Thermo Fisher Scientific (cat# 11965092)
DMEM medium	500 ml GibcoTM DMEM, high glucose, Thermo Fisher Scientific (cat# 11965092); 50 ml heat-inactivated fetal bovine serum (FCS), Sigma-Aldrich (cat# S0615); 2mM L-Glutamine, anprotec (cat# AC-AS-0001); 5 ml Penicillin-Streptomycin (Pen/Strep), anprotec (cat# AC-AB-0024)

Supplementary Table S8: List of cell culture antibodies

Reagent / Resources	Source	Identifier
Anti-mouse aCD3e	BD Biosciences	Cat#553058
Anti-mouse aCD28	Biolegend	Cat#102116

Supplementary Table S9: List of murine antibodies used for flow-cytometry

**Reagent / Resources**	**Source**	**Identifier**
Hamster monoclonal anti-mouse CD3, APC/Cyanine7	BioLegend	Cat#100329
Rat monoclonal anti-mouse CD4, BV 605	BioLegend	Cat#100451
Rat monoclonal anti-mouse CD8, BV 711	BioLegend	Cat#100747
Hamster monoclonal anti-mouse CD194, BV421	BioLegend	Cat#131217
Mouse BD Fc Block	BD Biosciences	Cat#553142
Rat monoclonal anti-mouse Ly-6C, PE-Cy7	BD Biosciences	Cat#560593
Rat monoclonal anti-mouse GR-1, PE	Miltenyi	Cat# 130-102-426
Rat monoclonal anti-mouse Ly-6G, APC	Miltenyi	Cat# 30-093-140
Rat monoclonal anti-mouse CD11b, APC-Fire	BioLegend	Cat# 101261
Zombie Aqua™ Fixable Viability Kit	BioLegend	Cat# 423101
Hamster monoclonal anti-mouse CD3e, PE-Cy7	BD Biosciences	Cat # 552774
Rat monoclonal anti-mouse NKG2D, PE	eBioscience	Cat# 12-5882-81
Rat monoclonal anti-mouse CD49b, FITC	BD Biosciences	Cat# 553857
Rat monoclonal anti-mouse CD335, PerCP 5.5	BioLegend	Cat# 137609
Mouse monoclonal anti-mouse CD45, Alexa Fluor(R) 700	BioLegend	Cat# 157615
Human monoclonal anti-mouse RT1B, APC	Miltenyi	Cat# 130-108-708
Fixable Viability Stain 575V	BD Biosciences	Cat# 565694

Supplementary Table 10: Primers used in human qPCR.

GENE (human)	Primer sequence [5'-3']
h*CYP27A1*	Forward Sequence: GTG CTG CCT TTC TGG AAG CGAT
	Reverse Sequence: TAG CCA GAC ACC TGG ATG CCAT
h*CYP7B1*	Forward Sequence: CAC CAG AGA ACA ATT GGA CAG CC
	Reverse Sequence: GCT ACC AAG TCT CCC TTT CGC A
h*PPIB*	Forward Sequence: AAC GCA GGC AAA GAC ACC AAC G
	Reverse Sequence: TCT GTC TTG GTG CTC TCC ACC T
h*NFkB p65*	Forward Sequence: TGA ACC GAA ACT CTG GCA GCT G
	Reverse Sequence: CAT CAG CTT GCG AAA AGG AGC C
h*AKT*	Forward Sequence: TGG ACT ACC TGC ACT CGG AGA A
	Reverse Sequence: GTG CCG CAA AAG GTC TTC ATG G
h*SNAIL*	Forward Sequence: TGC CCT CAA GAT GCA CAT CCG A
	Reverse Sequence: GGG ACA GGA GAA GGG CTT CTC
h*VIMENTIN*	Forward Sequence: AGG CAA AGC AGG AGT CCA CTG A
	Reverse Sequence: ATC TGG CGT TCC AGG GAC TCA T
h*RPL30*	Forward Sequence: CTGGTGTCCATCACTACAGTGG
	Reverse Sequence: CCAGTCTGTTCTGGCATGCTTC

Supplementary Table 11: Primers used in murine qPCR.

**GENE (mouse)**	**Primer sequence [5'-3']**
mCyp27a1	Forward Sequence: TCA GGA GAC CAT CGG CAC CTT T
	Reverse Sequence: CCA GTC ACT TCC TTG TGC AAG G
mCyp7b1	Forward Sequence: CGG AAA TCT TCG ATG CTC CAA AG
	Reverse Sequence: GCT TGT TCC GAG TCC AAA AGG C
mPpib	Forward Sequence: GCA GGC AAA GAC ACC AAT GGC T
	Reverse Sequence: TTG GTG CTC TCC ACC TTC CGT A
mNkfb p65	Forward Sequence: TCC TGT TCG AGT CTC CAT GCA G
	Reverse Sequence: GGT CTC ATA GGT CCT TTT GCG C
mAkt	Forward Sequence: GGA CTA CTT GCA CTC CGA GAA G
	Reverse Sequence: CAT AGT GGC ACC GTC CTT GAT C
mSnai1	Forward Sequence: TGT CTG CAC GAC CTG TGG AAA G
	Reverse Sequence: CTT CAC ATC CGA GTG GGT TTG G
mIl-6ra	Forward Sequence: TGC AGT TCC AGC TTC GAT ACC G
	Reverse Sequence: TGC TTC ACT CCT CGC AAG GCA T
mAcat1	Forward Sequence: GCA GGG AAG TTT GCC AGT GAG A
	Reverse Sequence: GAA CAC GGT CTT GAG CTT TGG C
mAbca1	Forward Sequence: GGA GCC TTT GTG GAA CTC TTC C
	Reverse Sequence: CGC TCT CTT CAG CCA CTT TGA G
mHmgcr	Forward Sequence: GCT CGT CTA CAG AAA CTC CAC G
	Reverse Sequence: GCT TCA GCA GTG CTT TCT CCG T
mFgf-2	Forward Sequence: AAG CGG CTC TAC TGC AAG AAC G
	Reverse Sequence: CCT TGA TAG ACA CAA CTC CTC TC
mFoxp3	Forward Sequence: CCT GGT TGT GAG AAG GTC TTC G
	Reverse Sequence: TGC TCC AGA GAC TGC ACC ACT T
mTbet	Forward Sequence: CCA CCT GTT GTG GTC CAA GTT C
	Reverse Sequence: CCA CAA ACA TCC TGT AAT GGC TTG
mRpl30	Forward Sequence: TCCTTGCCAACAACTGTCCAGC
	Reverse Sequence: TTTCCACACGCTGTGCCCAGTT

Supplementary figure legend:

Supplementary Figure S1:

(A) Lung CT image and illustration of regions from which samples were taken for analysis, tumoral (TU: solid tumor tissue) and the tumor-free control region (CTR: > 5cm away from the solid tumor). Peri-tumoral region (PT: 2 cm around tumor**)** was taken but not analyzed in this study. **(B)** Correlation of relative *CYP7B1*/*RPL30* mRNA expression level measured in the control/tumoral region correlated with tumor diameter of resected tumors of patients with LUAD; CTR (n =30, p = 0.1016, $R^2 = 0.0928$), TU (n = 28, p = 0.1298, $R^2 = 0.092$). **(C)** Correlation of relative *Vimentin*/*RPL30* mRNA expression level with relative *CYP27A1*/*RPL30* mRNA expression level measured in the control/tumoral region of patients with LUAD; CTR (n =16, p = <0.0001, $R^2 = 0.7705$), TU (n = 15, p = <0.0001, $R^2 = 0.7617$).

Correlations **(B, C)** are shown by using simple linear regression. The two-tailed Pearson correlation analysis was performed to get the r and p value for figure **(B, C)**.

Supplementary Figure S2:

Timelapse imaging of control and α-linolenic acid treated A549 migration assay. (A) Control images at timestamp 00h:00min:00s and after 48 hours. ImageJ2 was used to evaluate remigrated area. **(B)** α-LA treated images at timestamp 00h:00min:00s and after 48 hours. ImageJ2 was used to evaluate remigrated area.

Supplementary Figure S3:

Gut inflammation score of dietary groups in the murine model of LUAD. Cytokine analysis in murine serum.

(A) Gut inflammation score was rated semiquantitative by an expert pathologist from the Institute of Pathology at the University Hospital Erlangen in a blinded study. (SD, standard diet n=3; AD, arteriosclerosis diet n=5; OD, ω-3-rich diet n=5; ID, intervention diet n=7). P (AD *vs.*OD) = 0.0224; P (AD *vs.* ID) = 0.0086. **(B)** Murine serum IL-5 level [pg/ml] (SD, standard diet n=11; AD, arteriosclerosis diet n=16; OD, ω-3-rich diet n=12; ID, intervention diet n=16), **(C)** Murine serum IL-4 level [pg/ml] (SD, standard diet n=10; AD, arteriosclerosis diet n=15; OD, ω-3-rich diet n=13; ID, intervention diet n=15) and **(D)** Murine serum IL-6 level [pg/ml] (SD, standard diet n=8; AD, arteriosclerosis diet n=15; OD, ω-3-rich diet n=13; ID, intervention diet n=16). was measured with *LEGENDplex^{TM} MU Th Cytokine Panel (12-plex)*

One-way ANOVA was used for **(A)**. Data are shown as mean values ± s.e.m. *P,0.05; **P,0.01.

Supplementary Figure S4:

Representative flow cytometry gating strategies: (A) Serum and supernatant samples were collected and processed according to LEGENDplex™ MU Th Cytokine Panel (12-plex) w/ VbP V03 kit (Biolegend, Cat#741044) protocol (*See Materials and Methods*) and beads were then acquired in *BD FACSymphony^{TM} A1* and analyzed in *Kaluza Analysis 2.1 (Beckman Coulter)*. Measured beads were divided into population A and B depending on their size and granularity and were then subdivided into their corresponding population representing the specific cytokines. Mean fluorescence intensity of the PE channel was used for further analysis of the actual cytokine concentration. **(B)**Representative flow cytometry staining of a blood sample from a C57BL/6 mouse with tumor. Blood cells were stained with fluorochrome labeled antibodies for the identification of the different lymphocytes subsets. Cells were gated to identify CCR4+ CD4+ T regulatory cells. Therefore, doublets were excluded gating FSC-A against FSC-H. Afterwards, the lymphocytes are gated in FSC-A against SSC-A. Next, T cells were identified gating on CD3+ cells. Then, CD4+ and CD8+ T cells were identified gating on CD4 and CD8. To identify CCR4+ CD4+ cells, we gated CCR4+ cells on the CD4+ population versus the SSC-A.

Supplementary Figure S5:

mRNA expression levels of various genes measured in murine lung tissue samples. (A) *Hmgcr* mRNA expression in lung tissue samples of tumor bearing mice fed with standard diet (SD; n = 8), arteriosclerosis diet (AD; n =11), ω-3-rich diet (OD; n = 9) and intervention diet (ID; n = 12) in relation to the housekeeping gene *Rpl30*. P values as indicated. **(B)** *Acat1* mRNA expression in lung tissue samples of tumor bearing mice fed with standard diet (SD; n = 9), arteriosclerosis diet (AD; n =12), ω-3-rich diet (OD; n = 8) and intervention diet (ID; n = 11) in relation to housekeeping gene *Rpl30*. P values as indicated. **(C)** *Cyp27a1* mRNA expression in lung tissue samples of tumor bearing mice fed with arteriosclerosis diet (AD; n =11) compared to mice fed with ω-3-rich diet (OD; n = 10) in relation to housekeeping gene *Rpl30*. P (AD *vs.* OD) = 0.0446. **(D)** *Tbet* mRNA expression in lung tissue samples of tumor bearing mice fed with standard diet (SD; n = 8), arteriosclerosis diet (AD; n =12), ω-3-rich diet (OD; n = 9) and intervention diet (ID; n = 13) in relation to housekeeping gene *Rpl30*. P values as indicated. **(E)** *Ffg-2* mRNA expression in lung tissue samples of tumor bearing mice fed with standard diet (SD; n = 7), arteriosclerosis diet (AD; n =12), ω-3-rich diet (OD; n = 9) and intervention diet (ID; n = 12) in relation to housekeeping gene *Rpl30*. P values as indicated. **(F)** *Fgf-2* mRNA expression in lung tissue samples of tumor bearing mice fed with standard breeding diet (SD; n =7) compared to mice fed with ω-3-rich diet (OD; n = 9) in relation to housekeeping gene *Rpl30*. P value as indicated.

Kruskal-Wallis test was used for **(A; B; D; F)**. Mann-Whitney test was used for **(C)**. Welch's t test was used for **(E)**. Data are shown as mean values ± s.e.m.; *P,0.05.

Supp. Fig. 1

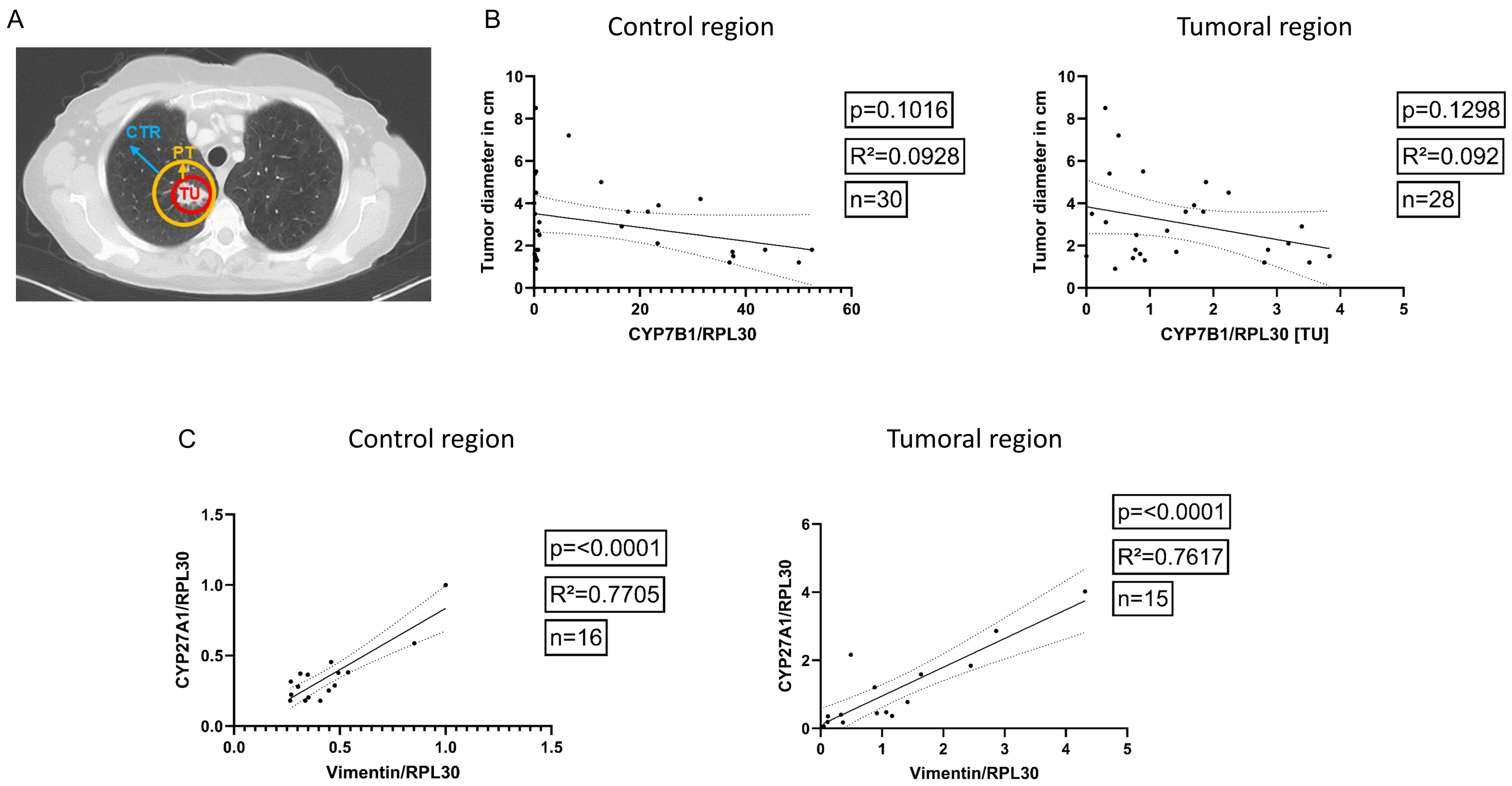

A
CTR
PT
TU
B
Control region
Tumor diameter in cm
CYP7B1/RPL30
p=0.1016
R²=0.0928
n=30
Tumoral region
Tumor diameter in cm
CYP7B1/RPL30 [TU]
p=0.1298
R²=0.092
n=28
C
Control region
CYP27A1/RPL30
Vimentin/RPL30
p=<0.0001
R²=0.7705
n=16
Tumoral region
CYP27A1/RPL30
Vimentin/RPL30
p=<0.0001
R²=0.7617
n=15

Supp. Fig. 2

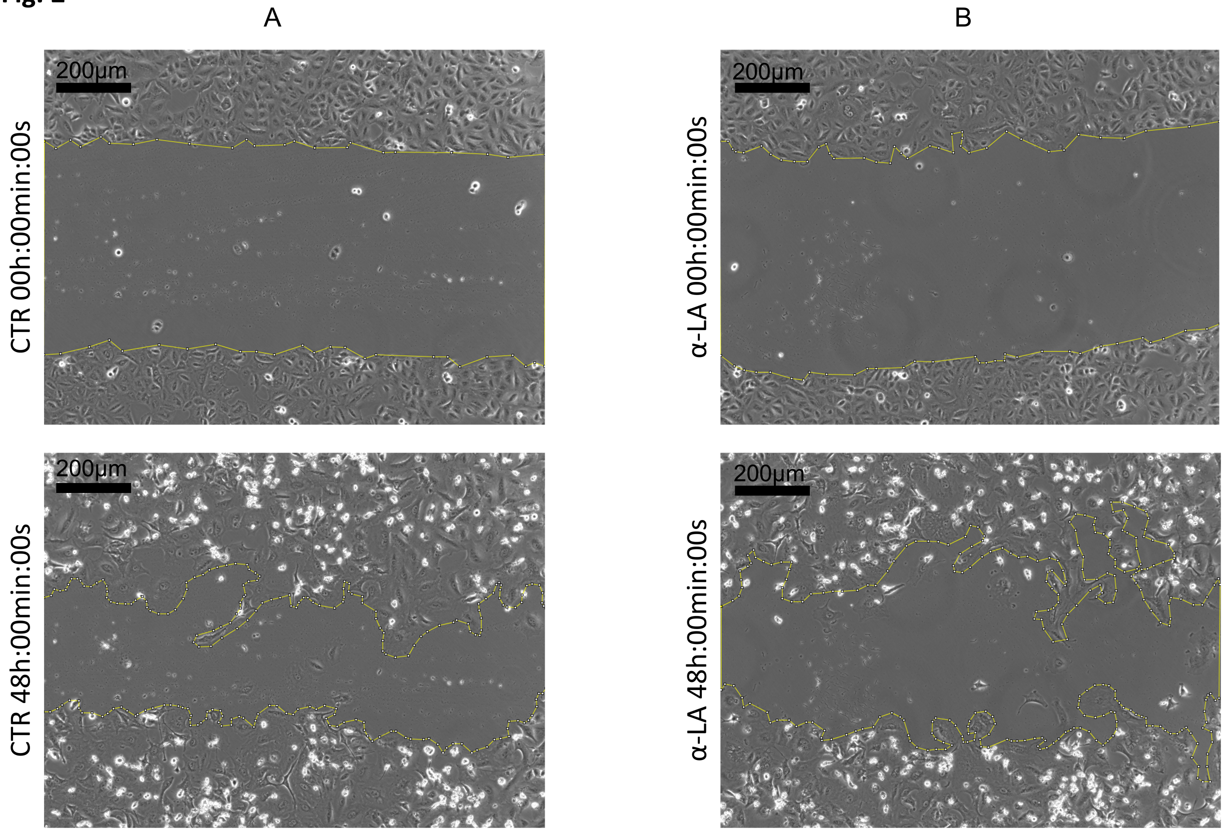

A
B
CTR 00h:00min:00s
200μm
α-LA 00h:00min:00s
200μm
CTR 48h:00min:00s
200μm
α-LA 48h:00min:00s
200μm

Supp. Fig. 3

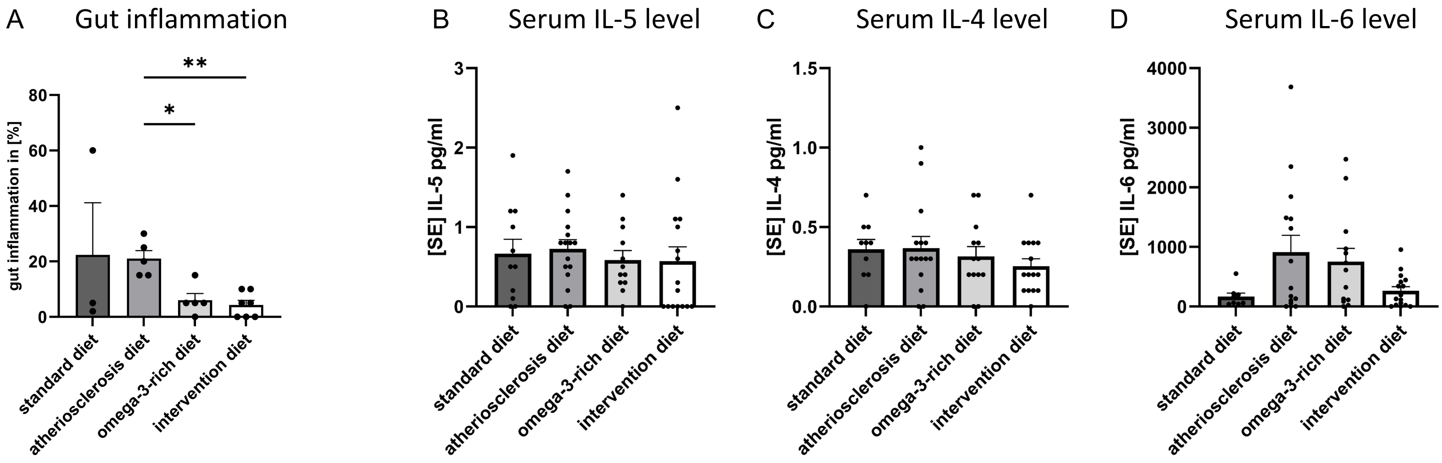
A
Gut inflammation
B
Serum IL-5 level
C
Serum IL-4 level
D
Serum IL-6 level
gut inflammation in [%]
[SE] IL-5 pg/ml
[SE] IL-4 pg/ml
[SE] IL-6 pg/ml
**
*
standard diet
atherioscleroisis diet
omega-3-rich diet
intervention diet

Supp. Fig. 4

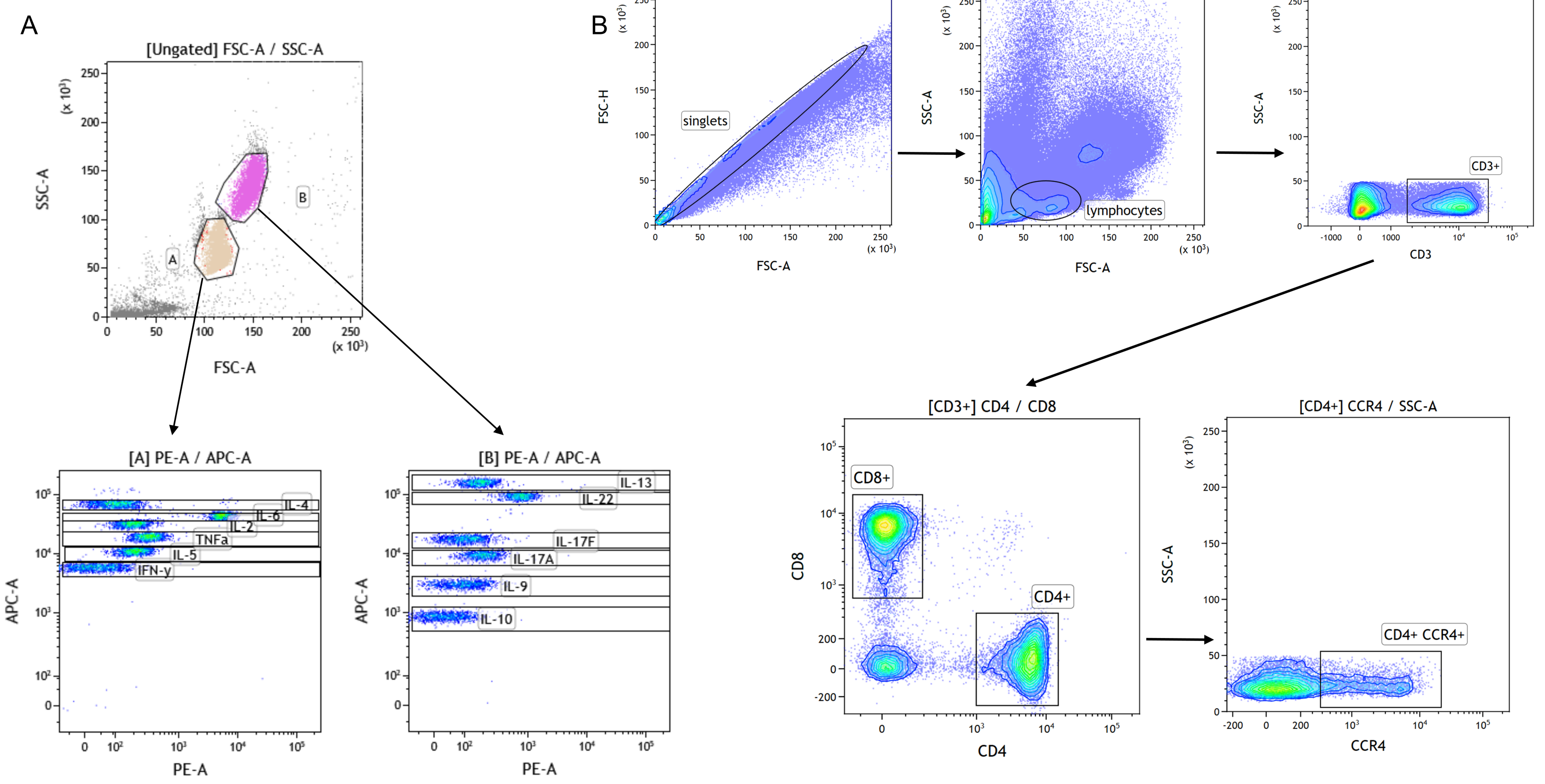
A
[Ungated] FSC-A / SSC-A
SSC-A
FSC-A
A
B
[A] PE-A / APC-A
IL-4
IL-6
IL-2
TNFa
IL-5
IFN-γ
APC-A
PE-A
[B] PE-A / APC-A
IL-13
IL-22
IL-17F
IL-17A
IL-9
IL-10
APC-A
PE-A
B
[Ungated] FSC-A / FSC-H
singlets
FSC-H
FSC-A
[singlets] FSC-A / SSC-A
lymphocytes
SSC-A
FSC-A
[lymphocytes] CD3 / SSC-A
CD3+
SSC-A
CD3
[CD3+] CD4 / CD8
CD8+
CD4+
CD8
CD4
[CD4+] CCR4 / SSC-A
CD4+ CCR4+
SSC-A
CCR4

Supp. Fig. 5

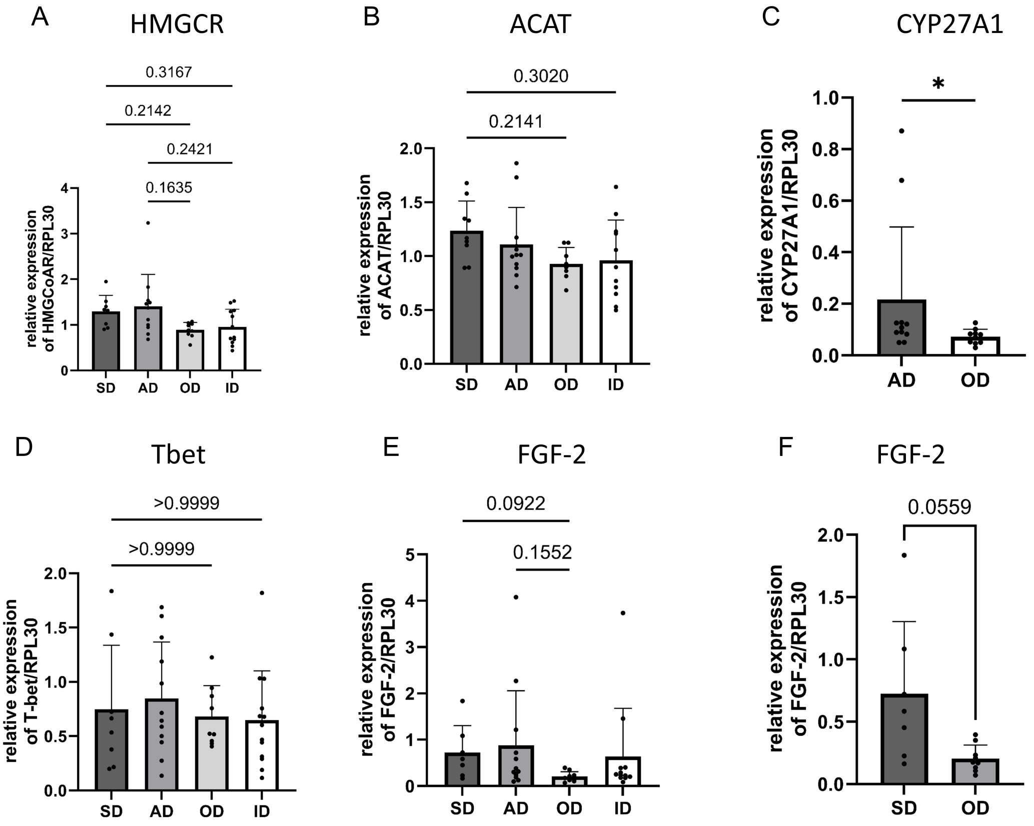
A
HMGCR
0.3167
0.2142
0.2421
0.1635
relative expression of HMGCoAR/RPL30
0
1
2
3
4
SD
AD
OD
ID
B
ACAT
0.3020
0.2141
relative expression of ACAT/RPL30
0.0
0.5
1.0
1.5
2.0
SD
AD
OD
ID
C
CYP27A1
*
relative expression of CYP27A1/RPL30
0.0
0.2
0.4
0.6
0.8
1.0
AD
OD
D
Tbet
>0.9999
>0.9999
relative expression of T-bet/RPL30
0.0
0.5
1.0
1.5
2.0
SD
AD
OD
ID
E
FGF-2
0.0922
0.1552
relative expression of FGF-2/RPL30
0
1
2
3
4
5
SD
AD
OD
ID
F
FGF-2
0.0559
relative expression of FGF-2/RPL30
0.0
0.5
1.0
1.5
2.0
SD
OD

Supp. Fig. 6

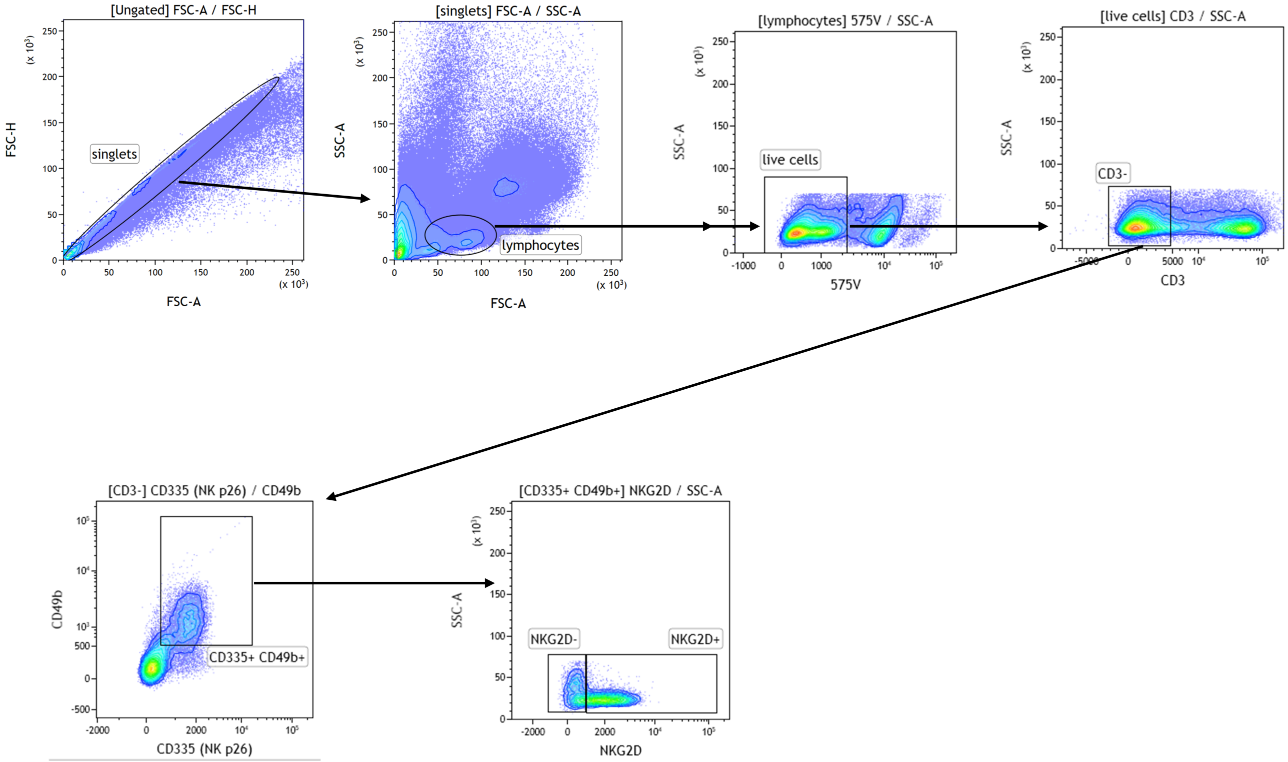
[Ungated] FSC-A / FSC-H
FSC-H
FSC-A
singlets
[singlets] FSC-A / SSC-A
SSC-A
FSC-A
lymphocytes
[lymphocytes] 575V / SSC-A
SSC-A
575V
live cells
[live cells] CD3 / SSC-A
SSC-A
CD3
CD3-
[CD3-] CD335 (NK p26) / CD49b
CD49b
CD335 (NK p26)
CD335+ CD49b+
[CD335+ CD49b+] NKG2D / SSC-A
SSC-A
NKG2D
NKG2D-
NKG2D+

Supp. Fig. 7

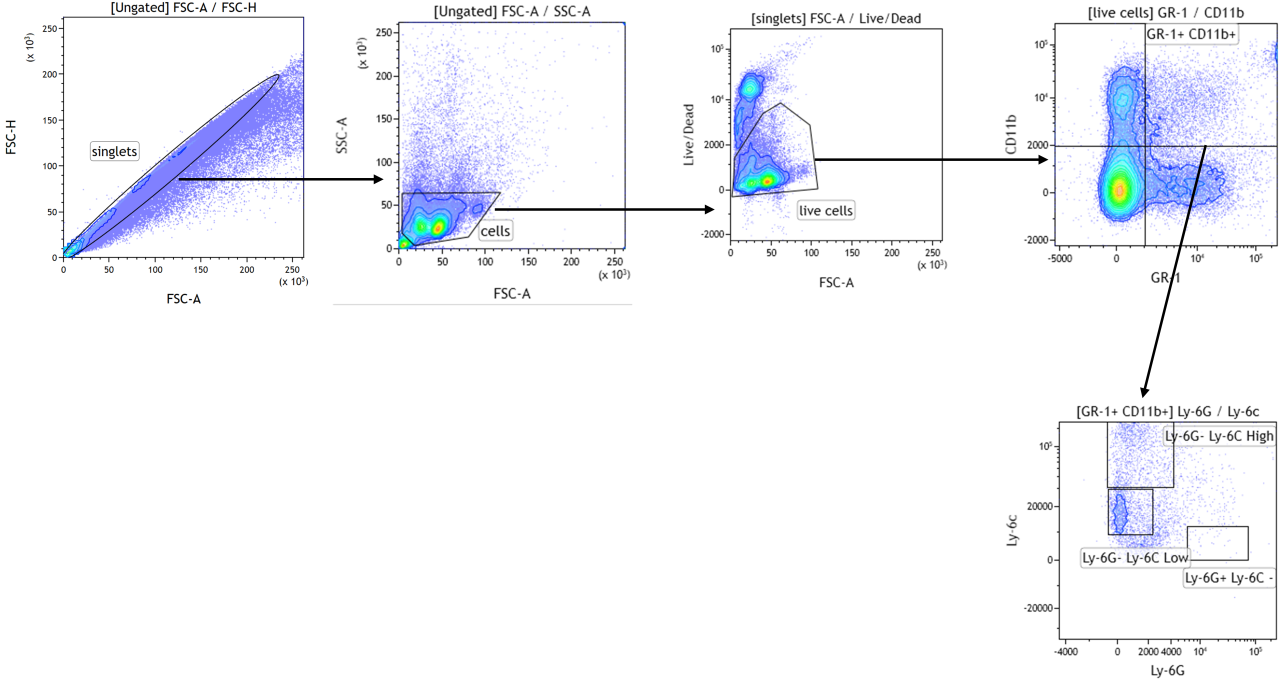

[Ungated] FSC-A / FSC-H
FSC-H
FSC-A
singlets
[Ungated] FSC-A / SSC-A
SSC-A
FSC-A
cells
[singlets] FSC-A / Live/Dead
Live/Dead
FSC-A
live cells
[live cells] GR-1 / CD11b
CD11b
GR-1
GR-1+ CD11b+
[GR-1+ CD11b+] Ly-6G / Ly-6c
Ly-6c
Ly-6G
Ly-6G- Ly-6C High
Ly-6G- Ly-6C Low
Ly-6G+ Ly-6C -
