# Supplementary material for: Dietary ω−3 polyunsaturated fatty acids (PUFAs) reduce cholesterol-driven non-small cell lung cancer (NSCLC) progression in mouse models of disease
Source: Commun Med (Lond). 2025 Oct 23;5:432. doi: 10.1038/s43856-025-01193-y (PMC12549838; doi:10.1038/s43856-025-01193-y)
Supplement: Supplementary file 3 — Description of Additional Supplementary Files [file 43856_2025_1193_MOESM3_ESM.pdf]

Description of Additional Supplementary Files

File name: Supplementary Data

Description: The numerical data plotted (source data) in Figures 1, 2, 3, 4, 5 and 6 as well as S3 and S5
